# Ten Simple Rules for Organizing an Unconference

**DOI:** 10.1371/journal.pcbi.1003905

**Published:** 2015-01-29

**Authors:** Aidan Budd, Holger Dinkel, Manuel Corpas, Jonathan C. Fuller, Laura Rubinat, Damien P. Devos, Pierre H. Khoueiry, Konrad U. Förstner, Fotis Georgatos, Francis Rowland, Malvika Sharan, Janos X. Binder, Tom Grace, Karyn Traphagen, Adam Gristwood, Natasha T. Wood

**Affiliations:** 1 Structural and Computational Biology (SCB) Unit, European Molecular Biology Laboratory (EMBL), Heidelberg, Germany; 2 The Genome Analysis Centre (TGAC), Norwich Research Park, Norwich, United Kingdom; 3 Heidelberg Institute for Theoretical Studies (HITS) gGmbH, Heidelberg, Germany; 4 Molecular Microbial Ecology Group, Institute of Aquatic Ecology, Universitat de Girona, Girona, Spain; 5 Centro Andaluz de Biología del Desarrollo (CABD), Universidad Pablo de Olavide, Sevilla, Spain; 6 Genome Biology (GB) Unit, European Molecular Biology Laboratory (EMBL), Heidelberg, Germany; 7 Core Unit Systems Medicine, University of Würzburg, Würzburg, Germany; 8 Luxembourg Centre for Systems Biomedicine (LCSB), University of Luxembourg, Luxembourg; 9 European Molecular Biology Laboratory, European Bioinformatics Institute (EMBL-EBI), Wellcome Trust Genome Campus, Hinxton, Cambridgeshire, United Kingdom; 10 Institute for Molecular Infection Biology, Universität Würzburg, Würzburg, Germany; 11 Bioinformatics Core Facility, Luxembourg Centre for Systems Biomedicine (LCSB), University of Luxembourg, Walferdange, Luxembourg; 12 Typeface & Lettering Design, Heidelberg, Germany; 13 ScienceOnline, Durham, North Carolina, United States of America; 14 Office of Information and Public Affairs, European Molecular Biology Laboratory (EMBL), Heidelberg, Germany; 15 South African National Bioinformatics Institute (SANBI), South African Medical Research Council Bioinformatics Unit, University of the Western Cape, Cape Town, South Africa; National Institutes of Health, United States of America

## Introduction

An academic conference is a traditional platform for researchers and professionals to network and learn about recent developments and trends in a particular academic field [1–4]. Typically, the organizing committees and sponsors decide the main theme and sub-topics of the conference and select the presenters based on peer-reviewed papers [5]. The selected speakers usually share their research with a large audience by means of presentations and posters. However, the most stimulating discussions generally take place over coffee breaks when attendees can interact with each other and discuss various topics, including their own research interests, in a more informal manner [1, 6, 7], while expanding their own professional networks. An emphasis on facilitating such informal/networking interactions is a central focus of “unconventional conferences”—or “unconferences.”

While many people may not yet have taken part in an unconference, the concept has been around for more than two decades. Events with unconference formats, beginning as early as 1985, include Open Space Technology, Foo Camp, BarCamp, Birds of a Feather, EdCamp, ScienceOnline, and many others. The success of these events has made the unconference format increasingly popular and widely known [8–11].

Unlike traditional conferences, an unconference is a participant-oriented meeting where the attendees decide on the agenda, discussion topics, workshops, and, often, even the time and venues. The informal and flexible program allows participants to suggest topics of their own interest and choose sessions accordingly. The format provides an excellent opportunity for researchers from diverse disciplines to work collaboratively on topics of common interest. The overarching goal for most unconferences is to prioritize conversation over presentation. In other words, the content for a session does not come from a select number of individuals at the front of the room, but is generated by all the attendees within the room, and, as such, every participant has an important role.

Advantages of the unconference format include: a focus on topics that are relevant to the attendees (because they suggested them), an opportunity for teamwork development, flexibility of schedule, and an emphasis on contributions from every participant. The relationships built during an unconference often continue well past the event. The interactions can lead to productive collaborations, professional development opportunities, and a network of resources and are very effective at building a community amongst participants. The unconference format, therefore, gives participants experience in working together, and this can change how they think about their day-to-day work.

A range of articles offer tips and advice for organizing and delivering aspects of scientific conferences and meetings or observations on features of successful meetings [5, 12, 13], including several from the *PLOS Computational Biology* “Ten Simple Rules” collection [14–16]. While the rules presented in this article are of particular relevance to the organization of unconferences, several of these points are also useful and complementary guidelines for organizing other kinds of events.

## Rule 1: How to Decide Whether to Run an Event As an Unconference or As a Traditional Conference

While there is no magic formula, reflecting on aspects such as participant numbers, venue size, expectations of attendees, and your overall objectives can be invaluable in deciding whether to run an event as an unconference or traditional conference. Unconferences are well suited to promoting interactions and networking between attendees as they allow a more flexible agenda. Discussion topics are shaped and influenced by participants, with exchanges of knowledge from many to many. This works particularly well when discussion groups are relatively small, creating a flexible, creative, and conducive environment for exchanges. A traditional conference, on the other hand, can be better suited to larger audiences, and when the focus of the meeting is more towards formal learning and knowledge sharing rather than involvement and interactions amongst participants. However, our experiences show that including unconference sessions in such events can be another valuable way of getting people involved, making connections, getting creative, achieving goals together, and developing a valuable platform for interactive knowledge exchange. It should also be noted that some successful unconferences are relatively large (e.g., ScienceOnline Together has 500 participants).

## Rule 2: Choose the Right Format

Depending on the mission and the goals of the participants, unconferences can be organized in many different ways. One example of an informal meeting is known as “Birds of a Feather”—these are events that usually accompany a traditional conference, where participants organize themselves to discuss topics without any pre-planned agenda, similar to “bar camps,” where the program is rewritten or overwritten on-the-fly by the participants using whiteboard schedule templates.

Other examples involving project-driven events include those mainly focused on technology topics and that involve software project development, such as “hackathons.” During such events, small sub-teams gather to work together on developing/addressing particular parts of a software project.

A little more organization is needed to arrange a “curated unconference” where topics and structures are collected by potential participants prior to the event. A group of organizers, in a transparent and open procedure, then sort through these ideas to build a structure of large and/or small-group discussion.

By forming smaller groups of participants to discuss different topics amongst each group, a “world café” style discussion allows participants to tackle several topics in a limited amount of time. At certain time intervals, every participant moves to a different table to participate in a specific discussion. Finally, all discuss the outcome of the different discussions under the moderation of the organizer.

In a “fishbowl” discussion, chairs are arranged in concentric circles with four to five chairs in the innermost circle (called the fishbowl), which channels the discussion as only participants in the fishbowl discuss the topic while others listen; participants wanting to join the vocal discussion approach the fishbowl and (via a mediator) replace one of the current members of the bowl.

Presentation styles at an unconference commonly include time limits, as exemplified by the “Ignite” and “Pecha Kucha” formats in which each presenter only has a very limited presentation time slot and slides advance automatically after 15 or 20 seconds, respectively. Such a format ensures that the presentations are succinct and fast-paced.

## Rule 3: Have a Clear Mission for the Meeting

Having a clear and visible mission statement can be a very effective way of focusing ideas for the content and structure of the event. It can turn collective minds to the development of a shared common goal that reduces emphasis on the individual and instead creates an event reflective of what the group needs and wants. From our experience, there are two major reasons why people attend unconferences: (1) to interact with many people of shared interests and (2) to learn useful information or skills related to their activities (often focused on their own career progression). A clear mission is a useful way of focusing the expectations of participants to the goals of the meeting. It can help to create an environment conducive to valuable and appropriate learning, and can guide discussions beyond a mere brainstorming session. Decisions about the focus and content of specific sessions become less subjective and remain transparent when the decision criteria align with the overarching goal of the meeting.

## Rule 4: Minimize the Lecture-Style Presentations

One of the defining features of an unconference is its inversion of the common features of more traditional meetings, in particular academic conferences. A common aspect of traditional meetings is the formal presentation (i.e., lecture style) with communication directed from one, typically a senior and powerful member of the community, to many others who listen passively and do not have much opportunity to actively interact with the presenter’s ideas. In contrast, unconferences typically minimize the use (and duration) of conventional presentations and prioritize cooperative knowledge. This means that the session content comes from the shared experiences and expertise of all participants in the room and not just from the front of the room. The idea that no individual person has all the answers promotes a spirit of generosity, interaction, and respect amongst all participants. Every voice is valued.

## Rule 5: Involve Participants in Planning and Structuring of the Event

Participant-centric thinking is perhaps the key feature that differentiates unconferences from more traditional meetings. Empowered participants, who know that they can directly influence and contribute to the structure and content of a meeting, tend to be much more invested in its success and outcome. However, the events still involve a certain amount of planning and infrastructure [14] and paying attention to details such as required equipment, venue, network connectivity, power outlets, and catering can have a large impact on the success of the event. Managing the flexibility of an unconference with appropriate logistical organization can avoid wasting time and, thus, avoid frustration for both the participants and organizers.

Participation is also where much of the enthusiasm and excitement of such meetings comes from, and there are many ways in which contributions can be facilitated. If a core group of organizers takes the lead in planning the event—including the program—then participants can focus on taking part in the discussion of ideas for sessions, content, or form of the unconference (see “Rule 2” for a variety of discussion formats and styles) instead of dealing with frustrating details. To ensure that the logistical arrangements are carried out prior to the event, the role of each organizer should be clearly communicated. As such, it may be beneficial to appoint one individual who coordinates the activities and is responsible for following-up on important preparations. Furthermore, the agenda should be visible to all participants before the unconference takes place and should include essential information such as the theme, sub-topics, time allowance, and contact information. These standard preparations allow the participants to arrive well informed and also create an opportunity for each participant to decide on how they may want to contribute to the unconference.

During the wrap-up of the event, any suggestions and feedback regarding the overall unconference events can be discussed and the theme of the next unconference can be decided. The goals of the next event will guide the planning and participants will be able to volunteer to be part of the new group of organizers. Finally, encouraging facilitators to include people who they know have interesting contributions to make ensures a core of contributors and promotes a lively discussion.

## Rule 6: Provide an Open, Relaxed Atmosphere

In order to make an unconference a success, the atmosphere of the event should be relaxed, open, friendly, and fun. This will ensure that all participants, especially those joining for the first time, feel welcome and respected. Creating and encouraging a casual and relaxed environment is favourable for everyone involved because it facilitates interaction and communication. To promote a relaxed atmosphere, think carefully about the layout of the venue. This includes the size of the room and the placement of tables and chairs; for example, arranging tables for small group discussions or placing chairs in a semi-circle or U-shape for group discussions. A good set-up not only fosters discussion but also has a positive impact on the overall quality of the unconference by strengthening the personal experience.

The organizers, as well as participants who have attended previous unconferences, should reach out and welcome newcomers to the format. By modelling conduct and values through their interactions with other participants both before and during the event (particularly at the start), they can strongly influence the way in which people interact with each other.

An effective way to encourage communication and participation is through ice-breaker activities during the early stages of the event. Small group activities are especially helpful since many participants may initially find it easier to interact actively in smaller, more intimate groups. This also helps new attendees meet new people and start to build relationships in a casual manner.

Fear of public speaking, questioning, and debating are common in all academic fields and communities. Unconferences aim to overcome these fears by creating an environment of respect that helps all participants gain self-confidence. Nominating capable, guiding facilitators who are able to ensure respectful communication throughout the meeting can achieve this goal. The facilitators should encourage all participants to share their own thoughts, listen to others’ comments, and—most importantly—consider all contributions. Repeating the name of a participant linked to a developed idea gives this participant a boost in self-confidence. However, in some cases, it may also mean that over-confident participants need to be “moderated” to provide enough time and space for the least confident participants to contribute voluntarily. Therefore, while diverse opinions are welcomed (and often result in stimulating discussions), the focus at an unconference is on how these different opinions are communicated. Good facilitators will create a natural atmosphere of mutual respect and trust.

## Rule 7: Trust Your Community

Unconferences prioritize focusing on, and engaging with, everyone who chooses to get involved in the event. This is in contrast to more traditional meetings, where the focus is much more on what the organizers have planned and the scheduled session presenters. Thus, in an unconference format, responsibility for the success of the event is more equally distributed across all participants. This shift of responsibility away from the organizers can initially seem intimidating, as it might seem like there are fewer ways to influence the success of the event. The lack of control can be difficult to accept, particularly for those who tend to micromanage. In an unconference format, the organizers will be successful if they trust the community to work with them to make the event a success. This power shift is worth embracing, rather than resisting, as it brings many exciting and energizing opportunities. Sharing leadership with the participants will create an atmosphere of personal empowerment, individual responsibility, and group ownership of the events.

This is perhaps not surprising; almost everyone choosing to participate in an unconference does so to personally benefit from the event. When given the chance to influence the success of the event, the attendees count this as a benefit in addition to the content of the unconference itself.

Another benefit is that the workload of an organizer may be reduced if it can be shared amongst a group of volunteers. Finally, trusting in the community makes it easier and less risky to experiment with novel formats and topics. Even when these experiments do not work out as planned, the very act of trying new ideas by involving, engaging, and trusting in participants brings the community closer together and delivers its own kind of success in terms of networking and community building. Learning to trust the community is key to embracing and enjoying the special character of these events.

## Rule 8: Communication Is Key to Your Event; Make it As Easy, Unambiguous, and Transparent As Possible

Engaging in communication is one of the reasons why people choose to come together for any meeting. One main characteristic of unconferences is the emphasis on interactive communication that gives all participants a chance to have their contributions heard by others. To this end, make use of multiple existing collaborative tools that assist in the communication before, during, and after an event. For example, a wiki can be very helpful in giving participants the chance to get involved in the organization of the event in advance—including idea and topic collections, scheduling sessions, taking care of the infrastructure of an event, as well as finding accommodation and ride shares for low-cost events.

Several tools exist to help with jotting down notes or minutes during a session: classic white boards and colored pens can be useful to collect suggestions and develop ideas together; even getting participants to scribble their thoughts down on paper tablecloths (which is a low-cost and low-tech collaborative tool with great haptic feedback) has proven to be handy and fun. The final work can be photographed and the pictures made available online later. Web-based collaborative real-time editors like Etherpad (http://etherpad.org/) can be helpful to conceptualize thoughts and to track discussions, as they can be edited by multiple people in parallel and can be used afterwards as an equivalent to conference proceedings. However, these Web-based editors require a working Internet connection throughout the event, which may not be practical at each event. Social media such as Twitter can also be utilized to share topics, progress, statements, or questions with people who are not present at the session. Here it is important to agree on a short, but distinctive, hashtag as soon as possible to enable people to follow and keep track of the tweets. A Tweetwall—a large screen or a projector displaying the most current tweets associated with the event’s hashtag—can also be entertaining and informative.

## Rule 9: The Journey Is As Important As Its Destination

A great way to extract the collective expertise, knowledge, and experience of attendees during unconference sessions is to encourage participants to identify and work together towards a common goal, and to document how they attempted to get there. Any given event will rarely provide the time needed to take a goal or project from beginning to end; however, we have seen unconferences serve as excellent ways of brainstorming, developing initial plans, creating the outline for a project, and gathering together a group of enthusiastic collaborators.

It is important to have tools that allow attendees to share the resources, ideas, and challenges of the session conversations. Documenting content can be an effective way to engage people and also to further the legacy of the unconference session beyond the confines of the room. Such an approach provides a way for participants to reflect on the collective learning and thinking that took place, as well as providing the means to evaluate the success of the discussion. It is unlikely within the time constraints of a session or single event that participants will come up with “the one final answer” to a particular problem or challenge. Therefore, providing a collaborative tool to record the development of ideas during the unconference session is important. The documentation of the session is a resource for reflecting on the work done, enabling participants to think about the issue in different ways, allowing others to see the progress of the discussion, establishing ideas for future events, and building a network of collaborators. In other words, the recording of the journey yields many benefits, even if you do not reach your final destination.

## Rule 10: No Idea Is Too Trivial

When a diverse group works together, some individuals will be good at big picture suggestions and others will emphasize details. Both are needed and both should be encouraged. While discussions of new ideas often begin at the conceptual level, contributions that may seem trivial or detail-oriented in the moment can also be important to a project’s ultimate success. Thus, to avoid missing out on important contributions, it is essential to include even the seemingly trivial remarks or ideas. A good way to do this is to write down all ideas and suggestions, so that later they can be sorted and considered. Do not rule out anything when it is first suggested because brainstorming becomes the most productive when any idea that comes to mind is communicated without prior judgment of its value. One person’s unusual idea may spark the way forward.

## Final Thoughts

There is not one “right” way to organize an unconference, but there are certainly things to be sure to include (and to avoid!) so that the event is as successful as possible. Perhaps the key is thinking of the event as “we” instead of “me.”

## Crowdsourcing the Writing of This Article

The authors wanted to base the opinions and advice provided in this article on experience of diverse unconferences. By doing this, rather than relying on the opinions of a small group of authors, we hoped that the content would be useful to a wider range of people. Thus, we crowdsourced the content by contacting organizers of a range of unconferences and similar events and inviting them to join us as authors. We also invited as authors all participants of a Birds of a Feather session focused unconference at the ISMB/ECCB 2013 meeting in Berlin, including also those who contributed to this session remotely via Twitter. Finally, we also invited all organizers of the Heidelberg Unseminars in Bioinformatics series of events [17] to join as authors, as several of the initiators of this article are members of that group.

We began the crowdsourcing by collecting a list of possible rules for the article via a git-controlled repository [18]. This list was then trimmed to reduce redundancy and overlap, and all authors voted to identify the initial set of ten rules to be included in the article. Small teams of authors collaborated to write content for each rule using a Piratenpad (https://www.piratenpad.de/), an online collaborative writing tool similar to an Etherpad. Native English speakers amongst the authors then processed this first draft to provide a common tone and language to the article. The resulting draft was then discussed by all authors, distributed as a Word document, and edits were implemented on the basis of this discussion by one of the authors until a consensus version of the text was agreed upon and submitted to the journal.

Authors are listed in the byline in the order in which they made edits to the manuscript.
